# Potentially functional variants of *MAP3K14* in the NF-κB signaling pathway genes predict survival of HBV-related hepatocellular carcinoma patients

**DOI:** 10.3389/fonc.2022.990160

**Published:** 2022-09-02

**Authors:** Qiongguang Huang, Yingchun Liu, Moqin Qiu, Qiuling Lin, Xueyan Wei, Zihan Zhou, Xiumei Liang, Runwei Li, Weiyi Chen, Xianguo Zhou, Hongping Yu

**Affiliations:** ^1^ School of Public Health, Guangxi Medical University, Nanning, China; ^2^ Department of Experimental Research, Guangxi Medical University Cancer Hospital, Nanning, China; ^3^ Department of Respiratory Oncology, Guangxi Medical University Cancer Hospital, Nanning, China; ^4^ Department of Clinical Research, Guangxi Medical University Cancer Hospital, Nanning, China; ^5^ Department of Cancer Prevention and Control, Guangxi Medical University Cancer Hospital, Nanning, China; ^6^ Department of Disease Process Management, Guangxi Medical University Cancer Hospital, Nanning, China; ^7^ Department of Occupational and Environmental Health, Indiana University, Bloomington, IN, United States; ^8^ Key Laboratory of Early Prevention and Treatment for Regional High Frequency Tumor, Guangxi Medical University, Ministry of Education, Nanning, China; ^9^ Key Cultivated Laboratory of Cancer Molecular Medicine, Health Commission of Guangxi Zhuang Autonomous Region, Nanning, China

**Keywords:** hepatocellular carcinoma, *MAP3K14*, single nucleotide polymorphism, variant, overall survival

## Abstract

**Background:**

The NF-κB signaling pathway plays an important role in associating inflammation with tumor development and progression. However, few studies have reported that roles of genetic variants of the NF-κB signaling pathway genes in survival of patients with HBV-related hepatocellular carcinoma (HBV-HCC), especially with regards to potentially functional SNPs.

**Methods:**

We used multivariate Cox proportional hazards regression to evaluate associations between 2,060 single nucleotide polymorphisms (SNPs) in 20 NF-κB signaling pathway genes and survival of 866 HBV-HCC patients, which were randomly split (1:1) into discovery and validation datasets. Expression quantitative trait loci (eQTL) analysis was conducted to identify associations between survival-associated SNPs and mRNA expression of corresponding genes. Furthermore, online database was used to assess mRNA expression of corresponding genes and survival. Finally, receiver operating characteristic (ROC) curves were used to assess the prediction accuracy of models integrating both clinical and genetic variables on HCC survival.

**Results:**

A total of 6 SNPs in *MAP3K14* remained significantly associated with OS of HBV-HCC patients (*P*<0.05, BFDP<0.8). Further eQTL analysis demonstrated that significant correlations between the rs2074292 (G>A) A allele was associated with higher mRNA expression levels of *MAP3K14* (*P*=0.044) in normal liver tissue, which was associated with worse survival of HBV-HCC patients. In the additive model, after adjusting for age, sex, smoking status, drinking status, AFP level, cirrhosis, embolus and BCLC stage, the combined dataset showed that HBV-HCC patients carrying the rs2074292 AA and GA genotypes (HR=1.71, 95%CI= 1.29-2.27, *P*=0.000) (HR=1.40, 95%CI=1.10-1.77, *P*=0.005) have worse OS than GG genotype, respectively. The addition of risk genotypes to the prediction models increased the AUC significantly from 71.15% to 73.11% (*P*=0.012) and from 72.55% to 74.21% (*P*=0.010) for 1-year and 3-year OS, respectively.

**Conclusion:**

Our study indicated that *MAP3K14* rs2074292 A allele may be a potential predictor of HBV-HCC survival, likely regulating *MAP3K14* mRNA expression.

## Introduction

Liver cancer is one of the most common malignant tumors derived from the digestive system, characterized by high incidence and mortality. According to GLOBOCAN 2020 data, the annual number of new cases and deaths of liver cancer ranks sixth and third among malignant tumors. Specifically, there were 410,000 new cases and 391,000 deaths in China, accounting for approximately 45.3% and 47.1% around the world, respectively (1); 85-90% of cases were hepatocellular carcinoma (HCC) (2). Despite advancements in HCC therapy recently, 5-year survival rate for HCC patients still remains stubbornly low due to the spread, metastases and high rate of recurrence (3, 4); and the 5-year survival rate is only 15.2% in China (5). Previous studies have shown that molecular and genetic factors may play a significant role in HCC progression and prognosis (6). Therefore, it was crucial to find potentially functional biomarkers that may predict the survival of HBV-related HCC patients for individualized therapies.

Single nucleotide polymorphisms (SNPs) were associated with gene expression and functions and thus can affect cancer development and patient survival (6). To date, several genome-wide association studies (GWASs) of HCC have discovered SNPs associated with cancer risk and survival (7, 8). However, the majority of SNPs did not reach the highly stringent genome-wide level of significance, ignoring the functional effects of other SNPs. The biological pathway-based approach was a hypothesis-driven method in the post GWAS era, which may avoid the nuisance of multiple tests to identify and examine functional consequences of novel loci, uncovering mechanisms that may underlie the observed associations (9).

NF-κB forms a family of transcription factors that played an important role in a variety of cellular functions, including cell proliferation, apoptosis, angiogenesis, immune response and cell adhesion (10, 11). Furthermore, the NF-κB signaling pathway can associate inflammation with tumor development and progression (11). Some studies have indicated that NF-κB can regulate the expression of pro-inflammatory genes like tumor necrosis factor (TNF), inducible NO-synthase (iNOS), interleukin-1 (IL-1), matrix metallopeptidase-9 (MMP-9), and many other chemokines (12). In addition, regulated by TNFα, Pim-2 proto-oncogene, serine/threonine kinase (*PIM2*) was frequently upregulated in HCC and associated with poor prognosis of HCC patients and tumor recurrence (13). Further studies revealed that *PIM2* could activate NF-κB signaling pathway through upregulating phosphorylation level of receptor-interacting protein kinase 2 (RIPK2) (13).

Currently, the roles of SNPs in the NF-κB signaling pathway genes and their functions related to HCC growth and progression are not entirely clear. Therefore, in the present study, we investigated associations between potentially functional genetic variants in the NF-κB signaling pathway genes and the survival of HBV-HCC patients in a two-stage analysis of genotyping datasets.

## Materials and methods

### Study populations

**Figure 1:**
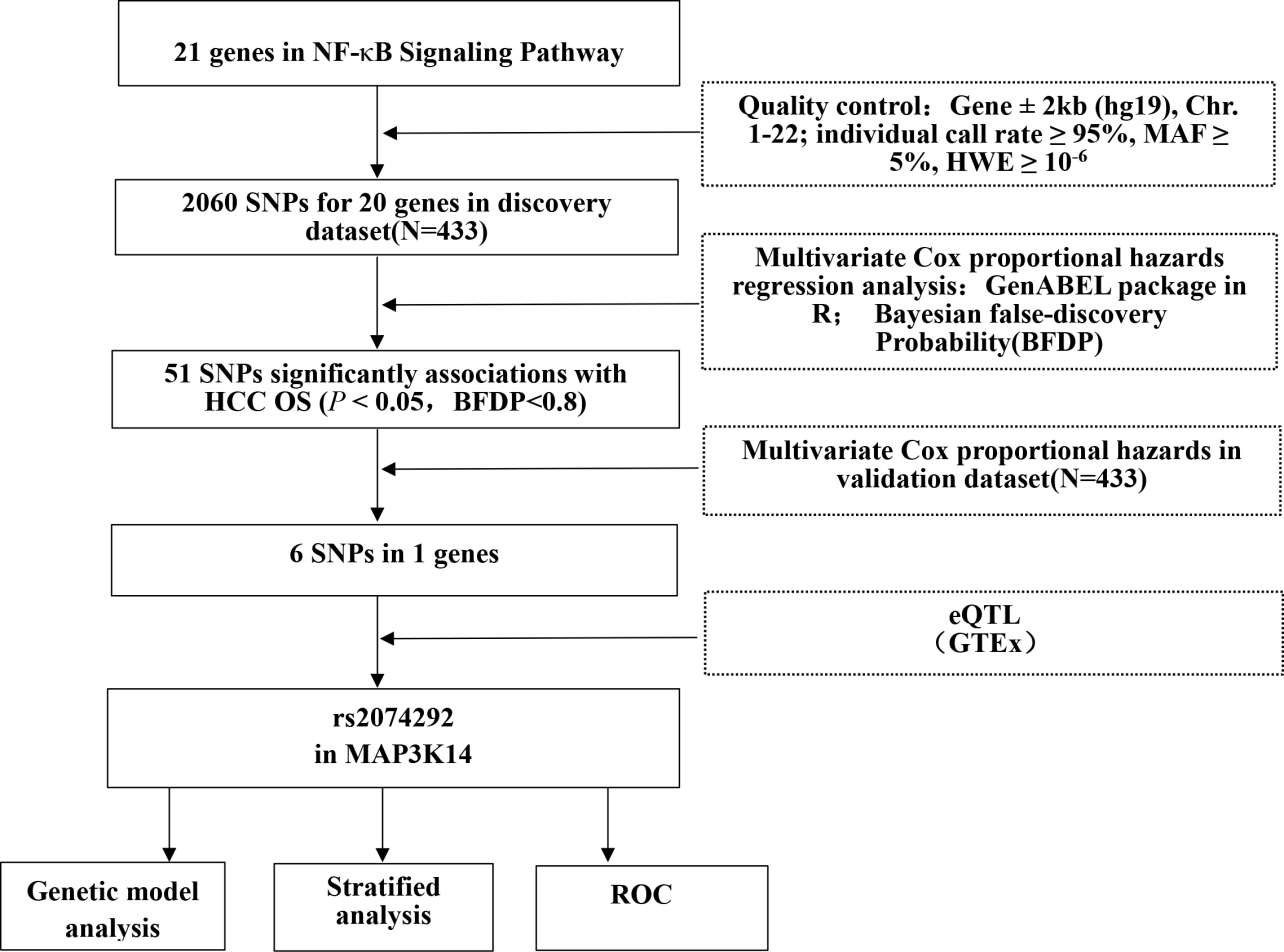
The flowchart of the present study. Abbreviations: SNP, single nucleotide polymorphism; ROC, receiver operating characteristic; OS, overall survival.

In this study, 1,063 patients with HBV-related HCC who underwent hepatectomy were recruited to build up the HCC Database. Among all participants, 877 patients were recruited from the Guangxi Medical University Cancer Hospital from July 2007 to December 2017, and 186 patients were recruited from the First Affiliated Hospital of Guangxi Medical University from June 2007 to December 2009. As shown in the study flowchart (**Figure 1**), a total of 866 HBV-HCC patients ultimately were recruited as the combined dataset for the study according to the following inclusion and exclusion criteria. Then, patients were randomized in a 1:1 manner to divide into discovery and validation datasets. Inclusion criteria: 1) For all the HCC patients, the diagnosis was made by pathological diagnosis, supported by angiography, sonography, computed tomography, alpha-fetoprotein (AFP) and/or magnetic resonance imaging; 2) All the HCC patients were seropositive for HBsAg. Exclusion criteria: 1) Patients with seropositive anti-HCV were excluded; 2) Patients with pathologic distant metastasis were eliminated; 3) Patients were excluded if they had cardiovascular or cerebrovascular events, renal dysfunction or pulmonary diseases; 4) Patients were excluded if they had received chemotherapy and/or radiotherapy before hepatectomy; 5) Patients who refused to follow-up or had no complete clinical information and follow-up data were excluded.

After signing a written informed consent, demographic data and relevant clinical information of participants were collected, such as age, sex, smoking status, drinking status, AFP level, cirrhosis, embolus and BCLC stage. In addition, 5 mL of peripheral blood was extracted from the patients, including 4 mL for DNA extraction and 1 mL to detect HBV infection status. All HCC patients were followed up every three months for the first two years of discharge after undergoing hepatectomy and every six months after two years. The content of the follow-up included subsequent treatment and death information, with overall survival (OS) as the outcome event, and the last follow-up time was in March 2020. Moreover, ethics committee approval and informed consent were obtained from the Guangxi Medical University Cancer Hospital (Approval Number: LW2022076).

### DNA extraction, genotyping and quality control (QC)

Genomic DNA was extracted from peripheral blood by using a blood DNA extraction kit (Concert, Xiamen, China) according to the protocol. Genotype detection of DNA samples was typed using an Illumina Infinium^®^ Global Screening Assay (Shanghai, China). Raw data was processed with PLINK (v1.9) for QC. SNP exclusion criteria were as follows: 1) sex chromosome SNPs; 2) a call rate < 95%; 3) minor allele frequency (MAF) < 0.1%; 4) Hardy-Weinberg equilibrium(HWE) *P* < 1×10^-6^. Samples exclusion criteria were as follows: 1) an overall genotyping call rate of ≤ 95%; 2) duplicates or first-degree relatives; 3) population outliers.

### Imputation

Based on the 1000 Genomes Project (Phase 3 v5) reference population information, Minimac3 was used for imputation (https://imputationserver.sph.umich.edu/index.htm) for the untyped SNPs. The inclusion criteria for imputation were as follows: 1) successfully genotyped in ≥ 95%; 2) Info score ≥ 0.3; 3) MAF ≥ 5%; 4) HWE *P* ≥ 1×10^-6^.

### Genes and SNPs selection

The genes involved in the NF-κB signaling pathway were selected by the Molecular Signatures Database (https://www.gsea-msigdb.org/gsea/msigdb/search.jsp) with the keyword “NF-κB.” After *IKBKG* in X chromosome was removed, 20 genes remained as candidate genes for further analysis (**Figure 1** and **Table S1**). We extracted all the SNPs in these genes and within their ± 2 kb flanking regions according to the following criteria: Individual call rate ≥ 95%, MAF ≥0.05, and HWE *P* ≥ 1×10^-6^. As a result, 2,060 genotyped SNPs were chosen from the dataset to further analysis.

### Statistical methods

Multivariate cox proportional hazards regression analysis was used to assess associations between SNPs of key genes in the NF-κB signaling pathway and HBV-related HCC OS (in an additive genetic model) in the discovery and validation datasets, with adjustment for age, sex, smoking status, drinking status, AFP level, cirrhosis, embolus and BCLC stage by using the GenABEL package of R software (14). We used the Bayesian false discovery probability (BFDP) method with a cut-off value of 0.80 for multiple testing correction to reduce the potential of false positive results (15). The risk or unfavorable genotypes was used to evaluate the effects of the identified SNP. Functional SNPs should be met all the following criteria: 1) the SNPs were associated with OS in discovery and validation datasets; 2) AS the eQTL analysis, genotypes of the SNPs were associated with mRNA expression of their genes from normal liver tissues in the genotype-tissue expression (GTEx) project(https://www.gtexportal.org/home/datasets) (9, 16). Then, bioinformatics prediction for the functional SNPs were performed with RegulomeDB (https://www.regulomedb.org/regulome-search/) and HaploReg v4.1 (https://pubs.broadinstitute.org/mammals/haploreg/haploreg.php) (17). Furthermore, the UALCAN database was used to analysis the mRNA expression levels of genes in tumor and normal tissues (http://ualcan.path.uab.edu/index.html), and the GEPIA database was used to detect the relationship between mRNA expression of genes and the survival of patients (OS and DFS) (http://gepia.cancer-pku.cn). Finally, receiver operating characteristic (ROC) curves were plotted, and time-dependent ROC analysis was evaluated to assess the prediction accuracy of models integrating both clinical and genetic variables on HCC survival using the “timeROC” package in R (version 4.0.3) (9, 18). Statistical analyses were performed by R software (4.0.3 and 3.1.3 versions), and *P*-values<0.05 were considered statistically significant.

## Results

### Associations of SNPs in the NF-κB signaling pathway genes with the survival of HBV-HCC patients

The basic characteristics of 866 HBV-HCC patients from the combined dataset have been described (**Table S2**). As the study flowchart is shown (**Figure 1**), a singlelocus multivariate cox proportional hazards regression analysis was first used to evaluate associations between 2,060 SNPs of NF-kB signaling pathway genes and HCC OS with adjustment for age, sex, smoking status, drinking status, AFP level, cirrhosis, embolus and BCLC stage in the discovery dataset. After multiple testing corrections by BFDP, 51 SNPs were identified to be significantly associated with HCC OS (*P* < 0.05, BFDP < 0.8). All of these significant associates were further validated by the validation dataset, and 6 SNPs in *MAP3K14* (mitogen-activated protein kinase kinase kinase 14) remained significantly associated with HCC OS (*P* < 0.05, BFDP < 0.8) (**Table 1**).

**Table 1:** Associations of six validated significant SNPs with HBV-HCC OS in discovery, validation and combined dataset.

SNP	Gene	Chr	Position	Discovery dataset			Validation dataset			Combined dataset		
				MAF	HR(95%CI)^a^	*P* ^a^ -value	MAF	HR(95%CI)^a^	*P* ^a^ -value	MAF	HR(95%CI)^a^	*P* ^a^ -value
rs2074292 G > A	*MAP3K14*	17	43361491	0.498	1.29(1.05-1.58)	0.015	0.441	1.31(1.08-1.60)	0.006	0.472	1.32(1.15-1.52)	7.96 × 10^-5^
rs11079502 A > G	*MAP3K14*	17	43350666	0.499	1.29(1.05-1.57)	0.015	0.450	1.35(1.11-1.65)	0.003	0.475	1.33(1.16-1.53)	6.66 × 10^-5^
rs2072090 G > C	*MAP3K14*	17	43351127	0.499	1.29(1.05-1.57)	0.015	0.450	1.35(1.11-1.64)	0.003	0.475	1.33(1.16-1.52)	7.14 × 10^-5^
rs2074291 C > A	*MAP3K14*	17	43361889	0.498	1.28(1.05-1.57)	0.015	0.442	1.32(1.08-1.60)	0.006	0.475	1.31(1.14-1.50)	1.37 × 10^-4^
rs9972936 G > A	*MAP3K14*	17	43353677	0.497	1.28(1.05-1.57)	0.016	0.448	1.34(1.10-1.63)	0.003	0.469	1.31(1.14-1.51)	1.20 × 10^-4^
rs4792842 G > A	*MAP3K14*	17	43360057	0.500	1.28(1.04-1.56)	0.018	0.450	1.32(1.09-1.60)	0.005	0.470	1.31(1.14-1.50)	1.32 × 10^-4^

a Multivariate Cox regression analyses were adjusted for age, sex, smoking, drinking, AFP, cirrhosis, embolus, BCLC.

MAF, minor allele frequency.

### The eQTL analysis

**Figure 2:**
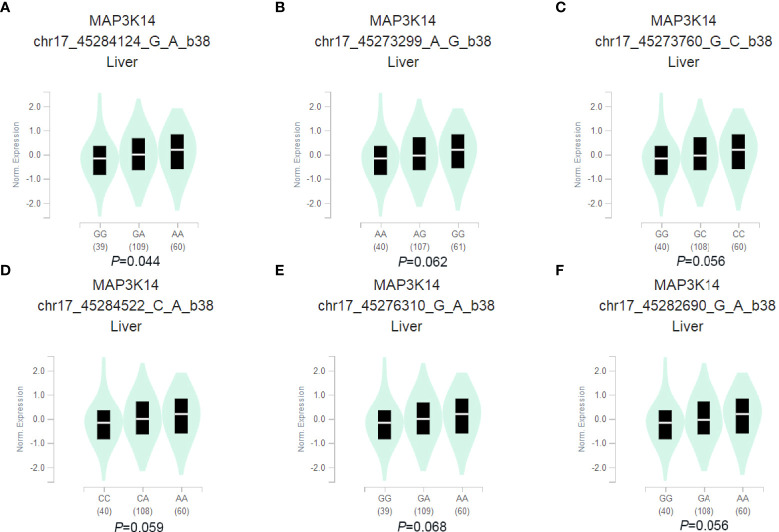
Correlation of SNPs with mRNA expression in normal liver tissue in the GTEx project. rs2074292 **(A)**, rs1107950 **(B)**, rs2072090 **(C)**, rs2074291 **(D)**, rs9972936 **(E)** and rs4792842 **(F)**.

In order to further explore the potential functions of the 6 SNPs, the eQTL analysis was performed to identify the correlations between genotypes of the SNPs and mRNA expression levels of their corresponding genes by using (GTEx) project. We found that the *MAP3K14* rs2074292 (G > A) A allele was associated with higher mRNA expression levels (*P*=0.044, **Figure 2A**) in normal liver tissue, and the eQTL of other five SNPs have no significant difference in normal liver tissues (**Figures 2B–F**). Furthermore, the online tools of RegulomeDB and Haploreg were used to predict the bioinformatics function of *MAP3K14* rs2074292. As a result, *MAP3K14* rs2074292 was found to have a significant impact on protein binding, motifs, chromatin structure, histone modifications and DNAse (**Table S3**).

### Differential mRNA expression analysis and survival of HBV-HCC patients

**Figure 3:**
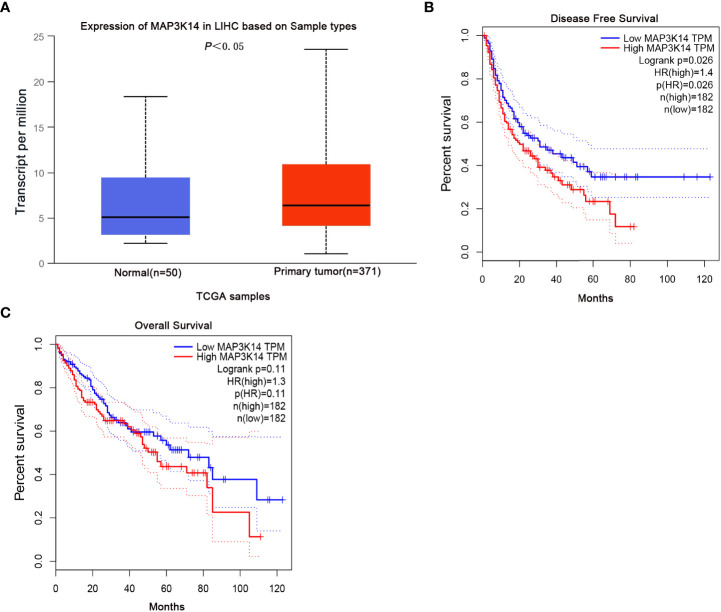
Differential mRNA expression analysis and survival of HCC from the UALCAN and GEPIA databases. Compared with normal tissues, *MAP3K14* mRNA expression was upregulated in tumor tissues (*P*<0.05) **(A)** and patients with high mRNA expression levels of *MAP3K14* had a worse DFS **(B)** but no significant difference in OS **(C)**.

To better understand the potential molecular mechanisms of the *MAP3K14* in the progression and survival of HCC, *MAP3K14* mRNA expression levels were evaluated in 371 primary tumor tissues and 50 normal tissue samples from the UALCAN database. As shown in **Figure 3**, compared with normal tissues, *MAP3K14* mRNA expression was upregulated in tumor tissues (*P*<0.05, **Figure 3A**). Additionally, we also assessed the correlation between *MAP3K14* mRNA expression level and survival of patients from the GEPIA and found that the patients with high mRNA expression levels of *MAP3K14* had a worse DFS (HR=1.4; Log-rank *P*=0.026, **Figure 3B**), but had no significant difference in OS of patients (HR=1.3; Log-rank *P* = 0.11, **Figure 3C**).

### Association of the functional SNP in the NF-κB signaling pathway with OS of HBV-HCC patients

In order to assess the associations between functional SNP with HBV-HCC OS, we performed a multivariate cox proportional hazards regression analysis adjusting for age, sex, smoking, drinking, AFP, cirrhosis, embolus, and BCLC under different genetic models (additive model, dominant model, and recessive model). It was found that HBV-HCC patients carrying rs2074292 AA genotypes (HR= 1.71, 95%CI=1.29-2.27, *P*=0.000) and GA(HR=1.40, 95%CI=1.10-1.77, *P*=0.005) had worse OS than GG genotype in the additive model. Furthermore, compared with rs2074292 GG genotypes in the dominant model, rs2074292 GA+AA genotypes (HR=1.48, 95%CI=1.18-1.85, *P*=0.001) had worse OS for HBV-HCC patients. In addition, rs2074292 AA genotypes (HR=1.38, 95%CI=1.10-1.74, *P*=0.006) were found to have a worse OS for HBV-HCC patients than rs2074292 GG+GA in the recessive model (**Table 2**).

**Table 2:** Associations between functional SNP and survival of HBV-HCC patients.

Genotype	Combined dataset(n=866)			
	All	Death	HR(95%CI)	*P^a^* -value
*MAP3K14* rs2074292 G>A				
Additive				
GG	236	105	1	
GA	447	219	1.40 (1.10-1.77)	0.005
AA	183	95	1.71 (1.29-2.27)	0.000
*P* trend				0.000
Dominant				
GG	236	105	1	
GA+AA	630	314	1.48 (1.18-1.85)	0.001
Recessive				
GG+GA	683	324	1	
AA	183	95	1.38 (1.10-1.74)	0.006

a Multivariate Cox regression analyses were adjusted for age, sex, smoking, drinking, AFP, cirrhosis, embolus, BCLC.

### Stratified analysis of the potentially functional SNPs in the datasets

All covariates were analyzed as categoric variables to further evaluate the association between risk genotypes and OS of HBV-HCC patients. As is shown in multivariate cox proportional hazards regression analysis, compared with 0 risk genotypes of HCC patients in multiple subgroups, HBV-HCC patients with 1 risk genotypes have increased the risk of death. No significant interactions between risk genotypes and each covariate on HBV-HCC survival were observed (**Table 3**).

**Table 3:** Stratification analysis of risk genotypes with OS of HBV-HCC patients.

Characteristics	0 risk genotypes		1 risk genotypes^a^			Multivariable analysis		
	All	Death		All	Death	HR (95%CI)	*P^b^ -*value	*P-*value for interaction
Age								0.245
≤ 47	120	56		314	177	1.66 (1.22-2.26)	0.001	
> 47	116	49		316	137	1.26 (0.91- 1.76)	0.164	
Sex								0.965
Female	26	8		80	34	2.01 (0.92-4.40)	0.080	
Male	210	97		550	280	1.44 (1.14-1.82)	0.002	
Smoking								0.959
NO	145	66		400	202	1.38 (1.04-1.83)	0.026	
Yes	91	39		230	112	1.69 (1.17-2.46)	0.006	
Drinking								0.596
NO	167	71		447	221	1.45 (1.10 -1.90)	0.007	
Yes	69	34		183	93	1.67 (1.11-2.52)	0.014	
AFP(ng/ml)								0.687
≤ 400	148	67		374	165	1.15 (0.86-1.53)	0.354	
> 400	88	38		256	149	2.00 (1.39-2.87)	<0.001	
Cirrhosis								0.361
NO	111	52		279	132	1.33 (0.96-1.84)	0.087	
Yes	125	53		351	182	1.64 (1.20-2.24)	0.002	
Embolus								0.927
NO	163	56		473	204	1.54 (1.14-2.07)	0.005	
Yes	73	49		157	110	1.43 (1.01-2.01)	0.043	
BCLC								0.655
0/A	107	32		320	114	1.30 (0.87-1.93)	0.196	
B/C	129	73		310	200	1.47 (1.12-1.93)	0.006	

a The low-risk group: patients carryed with 0 risk genotypes; The high-risk group: patients carryed with 1 risk genotypes(rs2074292 GA or AA);

b Multivariate Cox regression analyses were adjusted for age, sex, smoking, drinking, AFP, cirrhosis, embolus, BCLC.

### The ROC curves and time-dependent AUC

**Figure 4:**
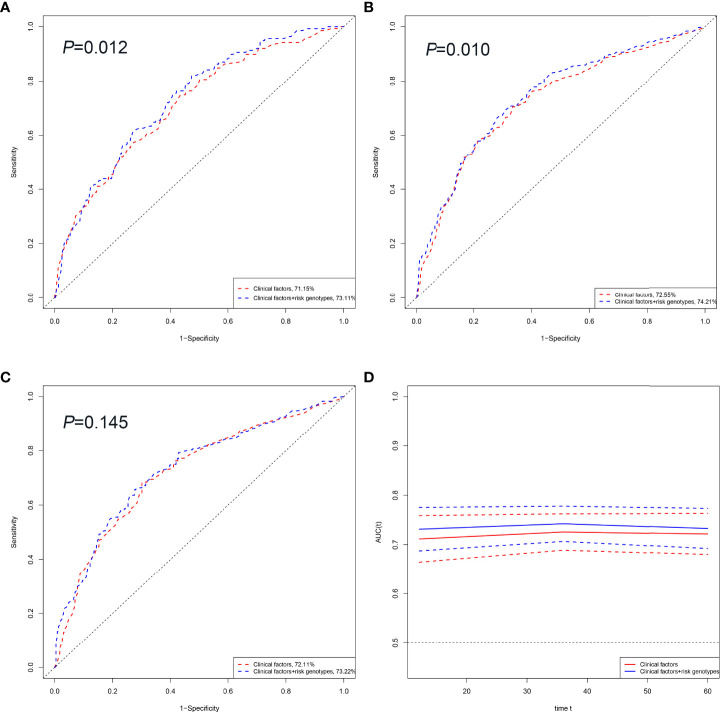
HBV-HCC survival prediction of the SNP by ROC curve in combined dataset. 1, 3 and 5-year HBV-HCC OS prediction by ROC curve **(A–C)**. Time-dependent AUC estimation for OS: based on age, sex, smoking, drinking, AFP, cirrhosis, embolus, BCLC and SNP **(D)**. Abbreviations: OS, overall survival ROC; receiver operating characteristic curve; AUC, area under curve.

To evaluate the predictive value of OS of HBV-HCC patients, we compared the area under the ROC curve (AUC) from the model with clinical variables to that from the model including both clinical variables and risk genotypes. The addition of risk genotypes to the prediction models increased the AUC significantly from 71.15% to 73.11% (*P*=0.012, **Figure 4A**) and from 72.55% to 74.21% (*P*= 0.010, **Figure 4B**) for 1-year and 3-year OS, respectively. However, the prediction performance of the model of 5-year OS increased the AUC from 72.11% to 73.22% without statistical significance (*P*=0.145, **Figure 4C**). Moreover, the time-dependent AUC curve suggested that including both clinical variables and risk genotypes could better predict the OS of HBV-HCC patients compared with using only clinical variables through the follow-up period (**Figure 4D**).

## Discussion

In this study, we first evaluated associations between 2,060 genetic variants of 20 genes in the NF-κB signaling pathway and HBV-HCC survival of 866 patients using a discovery dataset and validation dataset. It was found that 6 SNPs in *MAP3K14* remained significantly associated with OS of HBV-HCC patients. Expression quantitative trail loci analysis demonstrated that significant correlations between *MAP3K14* rs2074292(G>A) A allele were associated with higher mRNA expression levels (*P*=0.044) in normal liver tissue from the GTEx project, which was related to an increased risk of death from the GEPIA. Furthermore, multivariate cox proportional hazards regression analysis showed that HBV-HCC patients carrying the rs2074292 AA genotypes (HR= 1.71, 95%CI=1.29-2.27, *P*=0.000) and GA(HR=1.40, 95%CI=1.10-1.77, *P*=0.005) had worse OS than GG genotypes in the additive model. These results implied that the *MAP3K14* rs2074292(G>A) variant A alleles may play an essential role in the survival of HBV-HCC patients, possibly by regulating the mRNA expression levels of their related genes, and may provide critical biological evidence for the observed SNP-survival associations.

The mitogen-activated protein kinase kinase kinase 14 (MAP3K14), also known as NF-κB inducing kinase or NIK, was located on chromosome 17 in human, which played a central role in the noncanonical pathway (19, 20). Regulation of NIK activity occurs mostly at the post-translational level, and the overexpression of *NIK* was associated with metabolic disorders, inflammatory diseases and the development and progression of cancer (21). Meanwhile, The upregulated level of *NIK* mRNA was also essential in the number of breast cancer stem cells (CSCs) (21–23). *NIK* knockdown can reduce the expression of breast CSC markers, stem cell clonogenicity, and potential tumorigenic capacity (21, 23). Another study indicated that the positive expression rate of NIK in breast carcinoma tissue was significantly higher than that in normal tissue by immunohistochemistry and the 5-year survival of breast cancer patients with positive NIK expression was significantly lower than in those with negative NIK expression (24).

For HCC, it was reported that miR-520e expression was downregulated in HCC tissues and cell lines, and the introduction of miR-520e dramatically suppressed the growth of hepatoma cells (25). Further studies showed that miR-520e was directly bound to the 3’untranslated region of *NIK* to decrease *NIK* expression at the levels of mRNA and protein (25). These results showed that *NIK* has significant impacts on HCC tumorigenesis and progression.

In conclusion, we found that *MAP3K14* rs2074292(G>A) A allele was associated with worse OS for HBV-HCC patients and also associated with significantly higher mRNA expression levels of *MAP3K14* in liver tissues, which led to worse survival in HBV-HCC patients in the present study. Meanwhile, our results could provide new insight into the prognostic judgment and drug research and development of HBV-HCC patients. However, several limitations in this study should be discussed. Firstly, the study failed to collect all clinical information, which may affect judging the prognosis of HBV-HCC patients. Secondly, since the sample size of patients in this study was limited, studies with larger patient populations of various districts will be needed to validate our findings in the future as well. Finally, although our results indicated that *MAP3K14* rs2074292 played an important role in HBV-HCC survival and may serve as a potential therapeutic target for HBV-HCC, more experiments will need to performed to fully understand the underlying molecular mechanisms of *MAP3K14* rs2074292 in future studies.

## Data availability statement

The original contributions presented in the study are included in the article/**Supplementary Material**. Further inquiries can be directed to the corresponding authors.

## Ethics statement

The studies involving human participants were reviewed and approved by Guangxi Medical University Cancer Hospital. The patients/participants provided their written informed consent to participate in this study.

## Author contributions

Conception and design, HY, XZ, and QH. Administrative support, HY. Provision of study materials or patients, HY and XZ. Collection and assembly of data, ZZ and XL. Data analysis and interpretation, QH, YL, MQ, XW, and RL. All authors contributed to the article and approved the submitted version.

## Funding

This study was supported by the following grants: National Natural Science Foundation of China (81660567), The Key Research and Development Project of Guangxi (AB18050020), Key Project of Guangxi Natural Science Foundation (2018GXNSFDA050012), Shanghai Wu MengChao Medical Science Foundation (JJHXM-2019042), The Promoting Project of Basic Capacity for Young and Middle-aged University Teachers in Guangxi (2019KY0120), and Natural Science Foundation of Guangxi Province of China (2020GXNSFAA259022).

## Acknowledgments

The authors thank all the patients for their contributions to this study.

## Conflict of interest

The authors declare that the research was conducted in the absence of any commercial or financial relationships that could be construed as a potential conflict of interest.

## Publisher’s note

All claims expressed in this article are solely those of the authors and do not necessarily represent those of their affiliated organizations, or those of the publisher, the editors and the reviewers. Any product that may be evaluated in this article, or claim that may be made by its manufacturer, is not guaranteed or endorsed by the publisher.
