# Supplementary material for: Potentially functional variants of MAP3K14 in the NF-κB signaling pathway genes predict survival of HBV-related hepatocellular carcinoma patients
Source: Front Oncol. 2022 Sep 2;12:990160. doi: 10.3389/fonc.2022.990160 (PMC9478184; doi:10.3389/fonc.2022.990160)
Supplement: Supplementary file 1 [file Table_1.doc]

**Table S1. List of 20 selected genes in the NF-kB Pathway from MSigDB**

| **Dataset** | **Name of pathway** |  | **Selected genes^a^** | **Number of genes^b^** |
| --- | --- | --- | --- | --- |
| BIOCARTA | NF-κB_PATHWAY |  | *CHUK, FADD, IKBKB, IL1A, IL1R1, MAP3K1, MAP3K14, MAP3K7, MYD88, NFKB1, NFKBIA, IKBKG, RELA, RIPK1, TAB1, TNF, TNFAIP3, TNFRSF1A, TNFRSF1B, TRADD, TRAF6* | 20 |

^a^ Genes were selected based on MSigDB

^b^ *IKBKG* in X chromosome was removed

Keyword for MSigDB: NF-κB

Organism: Homo sapiens

**Table S2.** Associations of demographics and clinical characteristics with OS in HBV-related HBV-HCC patients from Combined dataset

| **Parameter** | **Combined dataset（n=866）** | | **MST**  **(Month)** | **Univariate analysis** | | **Multivariable analysis** | |
| --- | --- | --- | --- | --- | --- | --- | --- |
|  | **All** | **Death(%)** |  | **HR(95%CI)** | ***P -*value** | **HR(95%CI)** | ***P^a^ -*value** |
| Age |  |  |  |  |  |  |  |
| ≤ 47 | 434 | 233(53.69) | 47.00 | 1 |  | 1 |  |
| > 47 | 432 | 186(43.06) | 82.60 | 0.72(0.59-0.87) | <0.001 | 0.81(0.66-0.99) | 0.036 |
| Sex |  |  |  |  |  |  |  |
| Female | 106 | 42(39.62) | 66.97 | 1 |  | 1 |  |
| Male | 760 | 377(49.61) | 71.98 | 1.25(0.90-1.71) | 0.179 | 1.26(0.90-1.76) | 0.176 |
| Smoking |  |  |  |  |  |  |  |
| NO | 545 | 268(49.17) | 62.00 | 1 |  | 1 |  |
| Yes | 321 | 151(47.04) | 60.00 | 0.96(0.79-1.18) | 0.724 | 0.91(0.71-1.17) | 0.475 |
| Drinking |  |  |  |  |  |  |  |
| NO | 614 | 292(47.56) | 66.00 | 1 |  | 1 |  |
| Yes | 252 | 127(59.40) | 54.00 | 1.07(0.87-1.32) | 0.500 | 1.08(0.84-1.41) | 0.541 |
| AFP（ng/ml） |  |  |  |  |  |  |  |
| ≤400 | 522 | 232(44.44) | 70.30 | 1 |  | 1 |  |
| ＞400 | 344 | 187(54.36) | 39.10 | 1.59(1.31-1.93) | <0.001 | 1.29(1.05-1.57) | 0.015 |
| Cirrhosis |  |  |  |  |  |  |  |
| NO | 390 | 184(47.18) | 61.90 | 1 |  | 1 |  |
| Yes | 476 | 235(49.37) | 61.50 | 1.00(0.82-1.22) | 0.996 | 1.04(0.85-1.41) | 0.702 |
| Embolus |  |  |  |  |  |  |  |
| NO | 636 | 260(40.88) | 90.60 | 1 |  | 1 |  |
| Yes | 230 | 159(69.13) | 23.10 | 2.73(2.32-3.34) | <0.001 | 1.74(1.38-2.21) | <0.001 |
| BCLC |  |  |  |  |  |  |  |
| 0/A | 427 | 146(34.19) | 99.60 | 1 |  | 1 |  |
| B/C | 439 | 273(62.19) | 30.10 | 2.73(2.23-3.35) | <0.001 | 1.98(1.56-2.52) | <0.001 |

^a^ Multivariate Cox regression analyses were adjusted for age, sex, smoking, drinking, AFP, cirrhosis, embolus, BCLC.

Abbreviation: MST, median survival time.

**Table S3.** Function prediction of *MAP3K14* rs2074292

| **SNP** | **Gene** | **RegDB 2.0^a^** | |  | **Haploreg v4.1^b^** | | | |
| --- | --- | --- | --- | --- | --- | --- | --- | --- |
|  |  | **Rank** | **functional annotation** |  | **Promoter**  **histone marks** | **Proteins**  **bound** | **DNAse** | **dbSNP location** |
| rs2074292 | *MAP3K14* | 2b | Protein Binding, Motifs, Chromatin structure, Histone modifications |  |  |  | BLD,BLD | intronic |

^a^ RegulomeDB: https://www.regulomedb.org/regulome-search/

^b^ HaploReg: <https://pubs.broadinstitute.org/mammals/haploreg/haploreg.php>
